# Real-World Utility of GWAS-Based Diabetes Mellitus Panel Testing

**DOI:** 10.3390/ijms27010275

**Published:** 2025-12-26

**Authors:** In Hwa Jeong, Kyung-Won Hong, Ja-Eun Choi, Bo-Kyung Shine

**Affiliations:** 1 Department of Laboratory Medicine, Dong-A University College of Medicine, Dong-A University Hospital, Busan 49201, Republic of Korea; ihjeong@dau.ac.kr; 2 Theragen Health Co., Ltd., Seongnam 13488, Republic of Korea; kyungwon.hong@theragenhealth.com (K.-W.H.);; 3 Department of Family Medicine, Dong-A University College of Medicine, Dong-A University Hospital, Busan 49201, Republic of Korea

**Keywords:** genome-wide association study, Polygenic risk score, Type 2 diabetes mellitus, CDKAL1, *HHEX*, *KCNQ1*, *TCF7L2*, Real-world evidence

## Abstract

This study evaluated the clinical utility of a polygenic risk score (PRS)-based multigene panel test for predicting diabetes mellitus (DM) in a healthy population. A total of 302 individuals underwent genetic testing using the HelloGene™ DM panel, which includes four DM-related single nucleotide polymorphisms (*CDKAL1*, *HHEX*, *KCNQ1*, and *TCF7L2*). PRS values were calculated using an algorithm developed from the Korean Genome and Epidemiology Study (KoGES; n = 39,605), and participants were classified into four genetic risk groups (low, moderate, high, and very high). Fasting blood glucose, glycated hemoglobin (HbA1c), and body mass index were assessed at baseline and after at least three years of follow-up, and lifestyle factors including smoking, alcohol consumption, and exercise status were recorded. No significant differences in age, sex, or lifestyle habits were observed among PRS groups. The very high-risk group showed significantly higher follow-up fasting blood glucose levels (*p* = 0.001) and higher baseline and follow-up HbA1c levels (*p* = 0.0025 and *p* = 0.001, respectively), as well as a 4.5-fold increased risk of developing DM compared with other groups. Smoking significantly modified genetic risk, with smokers in the very high-risk group showing a 25% higher likelihood of developing DM. *CDKAL1* and *TCF7L2* variants were most prevalent in the moderate- and high-risk groups, while *HHEX* variants in the high-risk group showed the greatest susceptibility, particularly among current smokers. Overall, PRS-based genetic testing demonstrated potential clinical utility for stratifying individuals according to relative diabetes risk, highlighting a possible interaction between genetic susceptibility and lifestyle factors such as smoking

## 1. Introduction

Type 2 diabetes mellitus (T2DM) is a chronic metabolic disorder characterized by hyperglycemia and insulin resistance, which arises from a complex interplay of genetic, environmental, and behavioral factors [1]. The key contributors to T2DM include dietary habits, physical inactivity, obesity, sleep disturbances, and socioeconomic status. In South Korea, the prevalence of diabetes in 2022 was 12.5% among individuals aged 19 years and older and 14.8% among those aged 30 years and older. Although the age-standardized prevalence has remained relatively stable over the past decade, the absolute number of individuals with diabetes continues to increase due to population aging, and the projected prevalence is expected to rise substantially by 2051 [2]. This epidemiologic pattern underscores the need for effective strategies to identify high-risk individuals and implement targeted preventive measures.

Genetic predisposition plays a pivotal role in the development of T2DM, by contributing to both insulin resistance and beta-cell dysfunction [1]. Since the first genome-wide association study (GWAS) by the Wellcome Trust Case Control Consortium in 2007 [3], numerous studies have identified T2DM-associated genetic variants in various populations. Among these, four loci, *CDKAL1* (rs7754840), *HHEX* (rs1111875), *KCNQ1* (rs2237892), and *TCF7L2* (rs7903146) have consistently shown strong associations with T2DM in Western and Asian populations, including Koreans. Variants in the *CDKAL1* and *HHEX* loci have been associated with increased T2DM risk, with per-allele odds ratios typically reported in the range of 1.10–1.25, while *KCNQ1* and *TCF7L2* variants show comparable effect sizes across multiple ancestries. The *CDKAL1* locus has been linked to an increased risk of T2DM in the Japanese population [4], with C allele carriers exhibiting reduced first-phase insulin release and impaired proinsulin conversion [5,6]. Similar associations have been reported in Ashkenazi Jewish, African American, and Han Chinese populations [7,8,9]. Likewise, *HHEX* is significantly associated with T2DM in Caucasian, Japanese, and Korean populations, and has also been linked to impaired fasting glucose in Chinese Han individuals [10,11,12,13]. The *KCNQ1* locus strongly associated with insulin secretion following glucose intake and has been validated as a T2DM-associated gene in the Chinese and Korean populations [11,14,15]. Finally, *TCF7L2* is recognized as one of the most robust predictors of T2DM risk, with consistent associations across Japanese and Korean cohorts, including gestational diabetes cases [16,17,18].

Incorporating these genes into polygenic risk scores (PRS) offers a promising tool for stratifying individuals according to their genetic susceptibility to T2DM. By combining the effect sizes of multiple genetic variants into a single risk estimate, the PRS enables the identification of high-risk individuals and supports targeted prevention and personalized treatment strategies. Despite significant progress in understanding the genetic underpinnings of T2DM, an important gap persists in the assessment of the clinical utility of PRS in real-world settings. In particular, whether healthy individuals with high PRS are at a consistent risk of developing T2DM in healthy populations has not been thoroughly explored. This study aimed to address this gap by evaluating the predictive power of the PRS for diabetes onset, bridging the gap between genetic risk prediction and its practical implications for disease prevention and management.

## 2. Results

### 2.1. Selection of PRS Model

**Figure 1:**
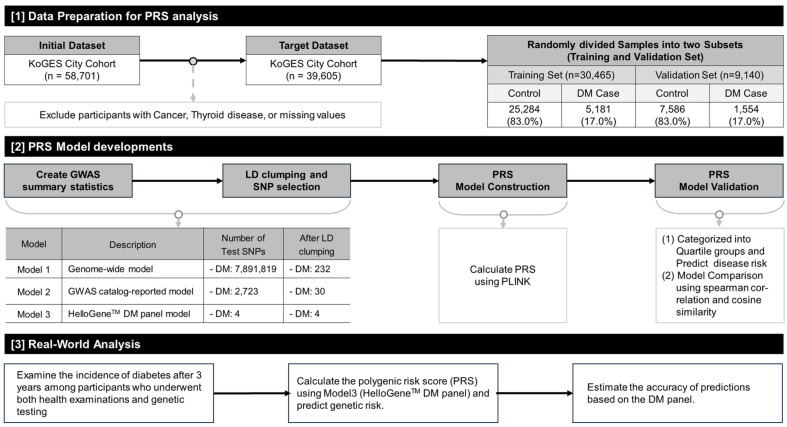
Schematic diagram of the study design, including data preparation, PRS model development process, and clinical analysis. Abbreviations: KoGES, Korean Genome and Epidemiology Study; PRS, Polygenic Risk Score; DM, Type 2 Diabetes Mellitus; GWAS, Genome-Wide Association Study; LD, Linkage Disequilibrium; PLINK, GWAS analysis software [19]. All arrows indicate the sequential workflow of the analysis.

Overall study design is described in Figure 1. To evaluate polygenic risk score models with different levels of genetic complexity and clinical applicability, we intentionally constructed three complementary PRS models. Model 1 represents a genome-wide approach incorporating a large number of variants to maximize population-level predictive performance. Model 2 reflects an ancestry-specific PRS based on SNPs identified in East Asian genome-wide association studies. Model 3 was designed as a targeted PRS using a limited number of well-established diabetes-associated SNPs, with the aim of assessing its potential utility in a clinically applicable genetic testing setting.

**Figure 2:**
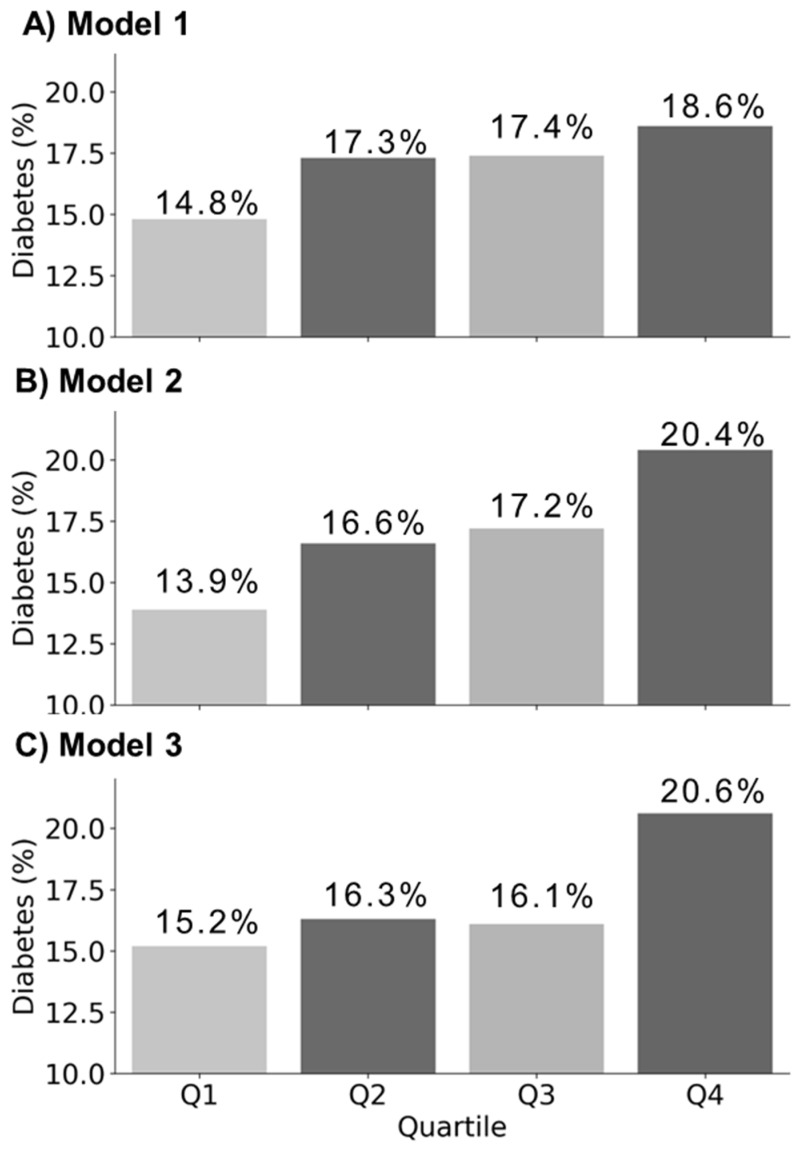
Comparison of diabetes case percentages across quartile groups in validation set (n = 9140, diabetes cases = 1554) for each PRS Model. (**A**) Model 1 is a genome-wide PRS model based on 7,891,819 SNPs; (**B**) Model 2 is an East Asian PRS model based on 2723 SNPs; (**C**) Model 3 is a HelloGene™ PRS model based on four SNPs identified by THERAGEN Health.

Using the KoGES dataset, three PRS models were developed to evaluate the diabetes risk, and the proportion of diabetes cases for each model is illustrated in Figure 2. The analysis showed that model 3 exhibited a relatively higher proportion of diabetes cases within the ‘Q4 risk quartile’ compared to models 1 and 2. The detailed distribution of diabetes cases across PRS quartile groups in the validation set is presented in Supplementary Table S2. Supplementary Figure S1 provides a comprehensive overview of the population characteristics of the KoGES cohort and the distribution of the PRS scores across the dataset. In Model 3, the targeted HelloGene™ PRS was calculated using beta coefficients estimated from the KoGES training set for the four selected diabetes-associated SNPs.

### 2.2. Baseline Characteristics of the Study Population

The study population included 258 males (85.4%) and 44 females (14.6%) with a median age of 48 years. The participants were categorized into four PRS risk groups: ‘low risk’ (59/302), ‘moderate risk’ (111/302), ‘high risk’ (70/302), and ‘very high risk’ (61/302). No significant differences were observed in age, sex, or habitual traits (e.g., smoking, alcohol consumption, and exercise habits) among the PRS categories. The follow-up FBG levels were significantly higher in the ‘very high risk’ group compared to that in the other groups (*p* = 0.001). The initial and follow-up HbA1c levels were also significantly higher in the ‘very high risk’ group compared to that in the other groups (*p* = 0.002 and 0.001, respectively) (Table 1).

### 2.3. Impact of HelloGene™ DM Panel to Total Study Population and ‘Current Smoker’ Group

**Figure 3:**
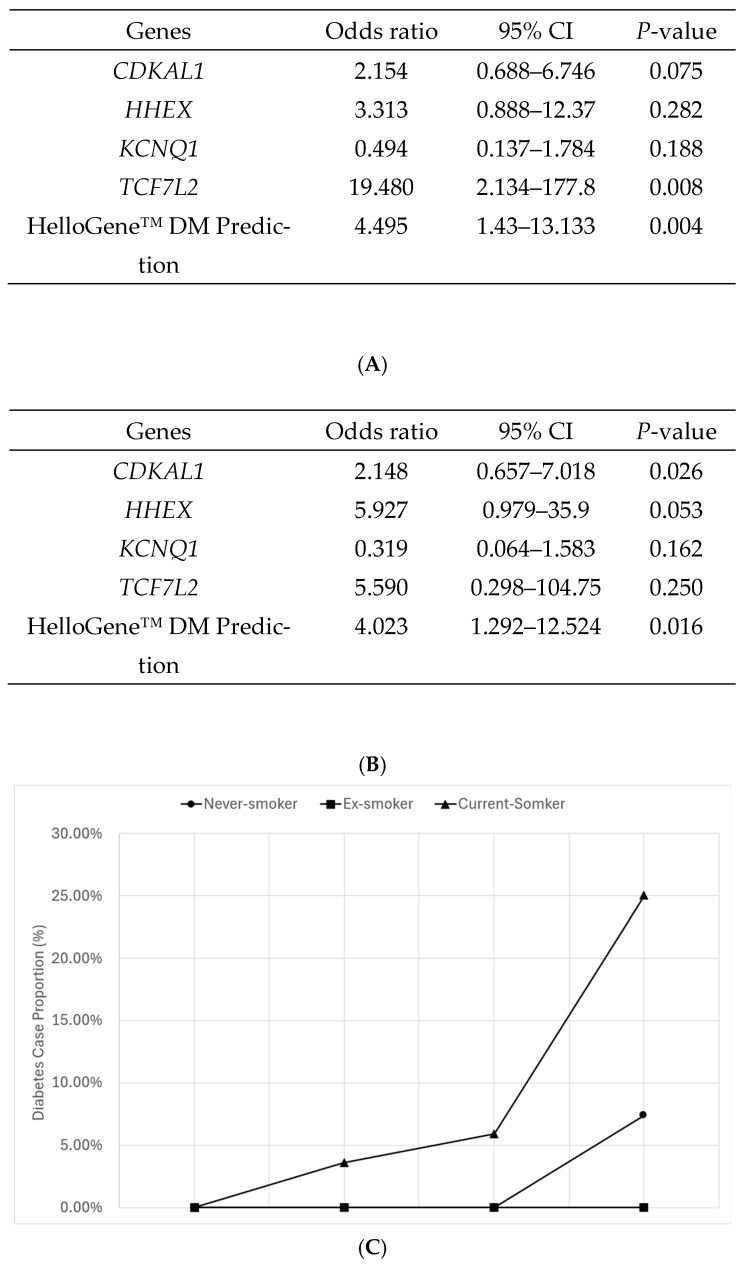
Results of logistic regression analysis adjusted for covariates including sex, age, smoking habits, alcohol consumption, exercise habits, and body mass index (BMI). The analysis assessed the correlation between the occurrence of diabetes by the HelloGene™ DM panel test, focusing on four diabetes-related genes (*CDKAL1*, *HHEX*, *KCNQ1*, and *TCF7L2*) as well as the comprehensive risk prediction based on the polygenic risk score (PRS) derived from these genes. (**A**) Impact of diabetes-related genotypes and prediction power of HelloGene™ DM panel test; (**B**) Impact of diabetes-related genotypes and prediction power of HelloGene™ DM panel test in the ‘current-smoker’ group; (**C**) Proportion of diabetes cases by smoking habits across HelloGene™ DM panel PRS quartile groups. Values represent observed proportions based on binomial data (number of cases/total number of individuals). Confidence intervals for odds ratio estimates are reported in the text and tables; conventional measures of variability (e.g., standard deviation or standard error) are not applicable to proportion data and were therefore not displayed.

The overall analysis showed that participants in the ‘very high risk’ HelloGene™ DM panel PRS quartile were approximately 4.5 times more likely to have diabetes compared to those in the lowest quartile (Odds ratio [OR] = 4.495, 95% confidence interval [CI]: 1.420–13.133, *p* = 0.004) (Figure 3A). A subgroup analysis based on the smoking status showed that the association was strongest among ‘current smokers,’ who were four times more likely to have diabetes than non-smokers (OR = 4.023, 95% CI: 1.292–12.524, *p* = 0.016), whereas the effect was less pronounced in other smoking groups (Figure 3B). Furthermore, when examining the proportion of diabetes cases by smoking habits across the HelloGene™ DM panel PRS quartile groups, approximately 25% of individuals in the ‘very high risk’ group who smoked were identified as having diabetes (Figure 3C).

The *CDKAL1* and *TCF7L2* genotypes were the most prevalent in moderate- and high-risk groups. Specifically, *CDKAL1* was predominant in the ‘high-risk’ group, while *TCF7L2* was most common in the ‘moderate risk’ group. Among current smokers, *HHEX* genotype carriers accounted for 25% of the total population (Supplementary Table S3). When the risk of developing DM was analyzed for each gene, only *TCF7L2* showed a statistical significance (*p* = 0.008). In the smoking subgroup, *HHEX* showed a borderline association with T2DM risk, which should be interpreted as an exploratory finding.

### 2.4. Clinical Utility of HelloGene™ Test

When moderate risk was selected for positive cutoff for clinical significance, the accuracy was 26.46% (95% CI: 21.57–31.82%). When high risk was selected for positive cutoff for clinical significance, the accuracy increased to 59.88% (95% CI: 54.12–65.46%). The selection of the very high-risk group as positive cutoff showed the strongest clinical significance: 80.48% accuracy with a 95% CI of 75.5–84.79% (Supplementary Table S4).

## 3. Discussion

This study evaluated the clinical utility of the HelloGene T2DM panel test in predicting T2DM risk in a real-world healthy population. By integrating PRS with follow-up metabolic and habitual data, we showed that a targeted PRS model based on four key T2DM-associated SNPs was able to stratify individuals into relative risk categories. The very high-risk group exhibited higher levels of metabolic markers and a greater proportion of diabetes cases during follow-up, suggesting relative enrichment of metabolic risk rather than definitive risk prediction.

The clinical utility of the PRS in patients with T2DM has been explored in several studies. In five cohorts encompassing diverse ancestries, T2DM PRS was significantly associated with the future development of T2DM in women with gestational diabetes mellitus [20]. In an Asian population, the integration of genome-wide PRS data with metabolic indicators, such as homeostatic model assessment of beta-cell function and insulin resistance, demonstrated enhanced predictive power, as shown in a study by Kang et al. [21]. Furthermore, the clinical applicability of T2DM PRS was examined in another East Asian cohort that identified its relevance in predicting the progression of glucose tolerance states, from normal glucose regulation to overt diabetes mellitus. We also conducted a more in-depth longitudinal follow-up to evaluate the predictive ability of T2DM in a larger cohort. Notably, individuals with T2DM in the top decile are more likely to be treated with insulin than those in the remaining PRS groups [22].

In our study, participants in the ‘very high-risk group’ exhibited significantly higher follow-up FBG and HbA1c levels. An association between smoking status and PRS scores was also observed, with a higher prevalence of T2DM among *HHEX* genotype carriers in the smoking subgroup. These findings suggest a potential combined influence of genetic susceptibility and lifestyle factors on disease risk, although formal statistical testing of gene–environment interactions was beyond the scope of the present study. Gene-specific analyses identified *TCF7L2* as the most significant predictor of T2DM in the overall population, confirming its well-established role as a robust genetic marker. In the smoking subgroup, *HHEX* showed a subgroup-specific and borderline association with T2DM risk, which should be interpreted as an exploratory finding. Together, these observations are consistent with prior evidence indicating that lifestyle modifications, such as smoking cessation, may help reduce disease risk in genetically susceptible individuals and warrant further investigation in studies specifically designed to evaluate gene–lifestyle relationships.

Clinical utility analysis showed that applying higher-risk cutoffs improved classification performance, with the ‘very high-risk’ group achieving an accuracy of 80.48% in identifying individuals who developed diabetes during follow-up. These findings reinforce the value of the PRS as a tool for identifying individuals who would benefit most from targeted interventions, such as intensive glucose monitoring or early lifestyle modifications. Importantly, the clinical utility discussed in this study refers to early risk stratification and identification of metabolic vulnerability in a real-world screening context, rather than long-term prediction of diabetes incidence, which would require substantially longer follow-up. However, the moderate accuracy at lower cutoff thresholds highlights the need to integrate the PRS with additional clinical and behavioral risk factors to further enhance risk classification performance.

Despite these promising findings, several limitations should be acknowledged. First, the real-world DONG-A-DM cohort was relatively small and derived from a single tertiary hospital–based health screening program, which may limit generalizability. In addition, the cohort was predominantly male and relatively young, reflecting the demographic characteristics of individuals undergoing voluntary health screening, and caution is therefore warranted when extrapolating these findings to women and older populations. Second, the follow-up duration of approximately three years, while sufficient to capture early metabolic changes and incident diabetes cases, may not fully reflect long-term T2DM risk. Third, important diabetes-related risk factors, including family history, detailed dietary patterns, precise physical activity measures, and socioeconomic status, were not comprehensively captured, and residual confounding cannot be excluded. Finally, subgroup analyses involving lifestyle factors were exploratory in nature and should be interpreted cautiously. These limitations highlight the need for larger and more diverse cohorts, as well as longer follow-up studies integrating comprehensive clinical, behavioral, and socioeconomic data.

Despite these limitations, this study provides meaningful evidence that a targeted 4-SNP PRS can stratify individuals into relative diabetes risk categories in a real-world health screening context. Rather than aiming for definitive risk prediction, the strength of this approach lies in its simplicity, interpretability, and feasibility for early risk stratification and identification of metabolic vulnerability. The observed enrichment of adverse metabolic profiles and diabetes cases in the very high-risk group supports the potential role of simplified PRS models as an initial screening tool to guide more intensive monitoring or lifestyle-focused interventions.

## 4. Materials and Methods

### 4.1. Study Design

The study population was divided into two groups: (1) the large-scale Korean cohort dataset from the Korean Genome and Epidemiology Study (KoGES), used to develop and validate the PRS for the HelloGene™ DM panel, and (2) the real-world dataset from Dong-A University Hospital (DONG-A-DM), used to evaluate the risk of diabetes onset and assess the clinical utility of the PRS. A schematic overview of the study methodology is shown in Figure 1. Detailed information on the KoGES samples, including sample size, demographic composition, and collection methods, is available in previous studies [23] and Supplementary Table S1.

### 4.2. Polygenic Risk Score Algorithm Construction

HelloGene™ Diabetes Mellitus Panel (THERAGEN Health Co., Ltd., Seongnam, Republic of Korea) was used to assess the genetic risk based on four SNPs: *CDKAL1* (rs7754840), *HHEX* (rs1111875), *TCF7L2* (rs7903146), and *KCNQ1* (rs2237892). PRS calculations were performed using Pruning and Thresholding (P+T) and Clumping and Thresholding (C+T) algorithms in the PLINK software (version 1.9) [19], with additional statistical analyses conducted using R software (version 4.2.2).

The KoGES dataset initially comprised 58,701 participants. However, after excluding individuals with cancer, thyroid disease, or missing data, the final sample size was 39,605 participants. Of these, 70% (30,465 participants) were allocated to the training set for the PRS model development, while 30% (9140 participants) were designated as the validation set. These exclusions were applied to minimize potential confounding, as cancer and thyroid disease—and their related treatments—can substantially influence glucose metabolism and insulin sensitivity, thereby obscuring the association between polygenic risk scores and type 2 diabetes risk.

Three PRS models were constructed for comparison: model 1 (genome-wide PRS based on 7,891,819 SNPs from GWAS summary statistics), model 2 (East Asian PRS derived from 2723 SNPs identified in East Asian GWAS) and model 3 (targeted PRS using four SNPs identified by THERAGEN Health). Linkage disequilibrium (LD) clumping was used to select independent SNPs, resulting in 232, 30, and 4 SNPs for models 1, 2, and 3, respectively. The PRS models were validated by applying them to the KoGES validation set, calculating individual PRS scores, and stratifying the participants into quartiles (Q1: low risk, Q2: moderate risk, Q3: high risk, and Q4: very high risk). The proportion of diabetes cases within each quartile was used to evaluate the predictive accuracy (Figure 2).

For each PRS model, individual PRS values were calculated as the weighted sum of risk alleles. The beta coefficients (β) used for polygenic risk score calculation represent log-odds effect sizes estimated from the KoGES training dataset (n = 30,465). These coefficients were derived using logistic regression models implemented in PLINK, with type 2 diabetes status as the outcome variable.

### 4.3. Study Subjects

The DONG-A-DM cohort was established as a retrospective observational cohort based on a routine health screening program at Dong-A University Hospital. The DONG-A-DM study population included 302 healthy individuals who underwent health examinations at the Dong-A University Hospital Health Examination Center between 2016 and 2021 and provided informed consent for genetic testing. As part of the routine health examination process at a tertiary university hospital, participants had previously provided informed consent for the use of their clinical, laboratory, and genetic data for research purposes. Although this consent was not restricted to diabetes-specific outcomes, all participants included in the present analysis were free of diabetes at baseline and were followed longitudinally to assess incident diabetes during follow-up. Participants returned for follow-up examinations 3 years after their baseline assessments, allowing for a longitudinal analysis of diabetes risk. Incident type 2 diabetes during follow-up was defined according to standard diagnostic criteria, including fasting plasma glucose ≥ 126 mg/dL, HbA1c ≥ 6.5%, physician-diagnosed diabetes, or initiation of antidiabetic medication, consistent with the American Diabetes Association and World Health Organization guidelines. Ethical approval was obtained from the Institutional Review Board (IRB) of Dong-A University (IRB No: DAUHIRB-EXP-24-212), and all participants provided written informed consent.

### 4.4. Clinical and Genomic Data Collection

Demographic and lifestyle data, including age, sex, smoking status, alcohol consumption, and exercise habits, were collected using structured questionnaires. The body mass index (BMI) was calculated using height and weight. Fasting blood glucose (FBG) levels were measured using the enzymatic UV method (AU5800, Beckman Coulter Inc., Brea, CA, USA), and glycated hemoglobin (HbA1c) levels were assessed using ion-exchange high-performance liquid chromatography (Tosoh HLC-723 G11 analyzer, Tosoh Corp., Tokyo, Japan).

Blood samples (3 mL) were collected in EDTA tubes for genetic testing. DNA was extracted from whole blood using the Exgene™ Blood Extraction SV kit (GeneAll, Seoul, Republic of Korea). Prior to genotyping, DNA concentration and purity were assessed using a Tecan F200 microplate reader (Tecan, Männedorf, Switzerland) to ensure quality. Genotyping was performed using the TaqMan assay on the OpenArray platform, adhering to the manufacturer’s protocol for the QuantStudio™ 12 K Flex Accufill System (Thermo Fisher Scientific, Waltham, MA, USA).

Genetic quality control procedures were applied prior to PRS construction. Samples with a genotyping call rate below 95% were excluded, and SNPs with call rates below 95% were removed. Hardy–Weinberg equilibrium testing was performed in the KoGES training dataset, and variants with significant deviation from equilibrium (*p* < 1 × 10^−6^) were excluded. These quality control steps were implemented to ensure robust and reliable genotype data for subsequent PRS analyses.

### 4.5. Statistical Analyses

Genetic data were coded 0 (non-risk allele homozygote), 1 (heterozygote), or 2 (risk allele homozygote). PRS was calculated as the weighted sum of risk alleles: ${PRS}_{j}=\sum_{i=1}^{N}\beta_{i}\;\times {dosage}_{ij}$ [24], where N represents the number of SNPs included in the score, *βi* is the log-odds effect size of variant *i* estimated from the KoGES training dataset, and dosage_*ij*_ is the number of risk alleles for variant *i* in individual *j*. PRS values were categorized into quartiles (Q1–Q4) based on their distribution in the KoGES dataset. The associations between PRS quartiles and diabetes risk were analyzed using ANOVA and logistic regression, adjusted for age, sex, smoking, alcohol consumption, and exercise. Interaction analyses stratified participants according to smoking status, alcohol consumption, and exercise status habit. Interaction and subgroup analyses stratified participants according to lifestyle factors were performed to explore potential effect modification.

A post hoc power analysis was conducted to evaluate whether the available sample size provided sufficient power to detect clinically meaningful differences in diabetes risk across PRS categories. Based on the observed incidence of diabetes, the study had adequate power to detect differences between the highest and lowest PRS quartiles. Primary analyses evaluating the association between PRS risk groups and diabetes outcomes were prespecified, whereas subgroup analyses according to lifestyle factors were considered exploratory. Accordingly, results from subgroup analyses were interpreted cautiously, with emphasis placed on effect size estimates and consistency of associations rather than strict multiple testing correction.

Statistical analyses were performed using SPSS version 18, with significance set at *p* < 0.05.

## 5. Conclusions

This study evaluated the performance of the HelloGene™ DM panel test for stratifying genetic risk of type 2 diabetes mellitus in a real-world health screening cohort. Using a targeted polygenic risk score based on four T2DM-associated SNPs, individuals were categorized into relative risk groups. Participants in the very high-risk group showed a higher prevalence of diabetes and less favorable metabolic profiles during follow-up, suggesting an enrichment of metabolic vulnerability. Overall, these findings indicate the feasibility of a simplified, disease-specific PRS approach for early risk stratification in routine screening settings. Although further large-scale and prospective studies are warranted to confirm long-term predictive value and broader clinical applicability, the present results provide preliminary evidence supporting the potential role of targeted PRS models in diabetes risk assessment.

## Figures and Tables

**Table 1:** Population characteristics of the study population and metabolic and habitual variables categorized by genetic test results.

Variables	Unit	Description	Total	HelloGene™ T2DM Genetic Test Results				Chi-Square or ANOVA
				Low Risk	Moderate Risk	High Risk	Very High Risk	*p*-Value
Sex	Male	n (freq)	258 (0.854)	50 (0.847)	97 (0.874)	58 (0.829)	53 (0.855)	0.865
	Female	n (freq)	44 (0.146)	9 (0.153)	14 (0.126)	12 (0.171)	9 (0.145)	
Age	Years old	mean ± SD	48 ± 7	49 ± 7	48 ± 8	48 ± 6	47 ± 6	0.669
BMI	kg/m^2^	mean ± SD	24.8 ± 3.0	25.0 ± 2.6	24.6 ± 2.8	25.1 ± 3.4	24.6 ± 3.2	0.616
Smoking	Never	n (freq)	122 (0.404)	23 (0.39)	44 (0.396)	28 (0.4)	27 (0.435)	0.997
	Ex	n (freq)	104 (0.344)	21 (0.356)	39 (0.351)	25 (0.357)	19 (0.306)	
	Current	n (freq)	76 (0.252)	15 (0.254)	28 (0.252)	17 (0.243)	16 (0.258)	
Alcohol consumption	Never	n (freq)	117 (0.387)	20 (0.339)	42 (0.378)	28 (0.4)	27 (0.435)	0.736
	Current	n (freq)	185 (0.613)	39 (0.661)	69 (0.622)	42 (0.6)	35 (0.565)	
Exercise habits	No	n (freq)	43 (0.142)	7 (0.119)	14 (0.126)	12 (0.171)	10 (0.161)	0.758
	Yes	n (freq)	259 (0.858)	52 (0.881)	97 (0.874)	58 (0.829)	52 (0.839)	
Baseline FBG	mg/dL	mean ± SD	98 ± 18	96 ± 17	97 ± 18	96 ± 16	103 ± 21	0.061
Follow-up FBG	mg/dL	mean ± SD	100 ± 19	96 ± 11	98 ± 16	98 ± 15	109 ± 30	**0.001**
Difference between baseline and follow-up FBG	mg/dL	mean ± SD	2 ± 16	0 ± 16	0 ± 13	2 ± 11	5 ± 24	0.233
Baseline HbA1c	%	mean ± SD	5.7 ± 0.8	5.5 ± 0.4	5.6 ± 0.8	5.6 ± 0.7	5.9 ± 1	0.025
Follow-up HbA1c	%	mean ± SD	5.8 ± 0.8	5.6 ± 0.4	5.6 ± 0.5	5.8 ± 0.9	6.2 ± 1.3	**0.001**
Difference between baseline and follow-up HbA1c levels	%	mean ± SD	0.1 ± 0.6	0.1 ± 0.3	0 ± 0.6	0.2 ± 0.6	0.2 ± 0.8	0.098
T2DM	Follow-up normal	n (freq)	294 (0.974)	59 (1)	110 (0.991)	69 (0.986)	56 (0.903)	**0.002**
	Follow-up DM cases	n (freq)	8 (0.026)	0 (0)	1 (0.009)	1 (0.014)	6 (0.097)	

The *p*-value of the variables that showed statistical significance is underlined in bold. Abbreviations: BMI, body mass index; FBG, fasting blood glucose; SD, standard deviation; T2DM, type 2 diabetes mellitus.

## Data Availability

The data presented in this study are hospital-based data managed by Dong-A University Hospital through its medical information system. The data are available from the corresponding author upon reasonable request, subject to institutional approval, but are not publicly available due to ethical and privacy restrictions.

## Supplementary Materials

The following supporting information can be downloaded at: https://www.mdpi.com/article/10.3390/ijms27010275/s1. References [25,26,27] are cited in the Supplementary Materials.
