# Supplementary material for: Real-World Utility of GWAS-Based Diabetes Mellitus Panel Testing
Source: Int J Mol Sci. 2025 Dec 26;27(1):275. doi: 10.3390/ijms27010275 (PMC12785355; doi:10.3390/ijms27010275)
Supplement: Supplementary file 1 [file ijms-27-00275-s001.zip › ijms-4036328-supplementary.pdf]

Supplementary Materials

1. Study Population and Design

The participants for this study were selected from the Korea Genome Epidemiology Study (KoGES), a government-funded platform designed to explore the genetic and environmental factors contributing to common chronic diseases in Korean adults. The study was initiated by the National Research Institute of Health, Ministry of Health and Welfare, and the Centers for Disease Control and Prevention. The KoGES recruited community-dwelling individuals and participants aged over 40 years at baseline from the National Health Examinee Registry. The details of KoGES have been described previously described [1].

2. Genotyping and Covariates

Genomic DNA was extracted from peripheral blood samples and genotyped using Korea Biobank Arrays (KoreanChip). The detailed methodology of the Korean chip has been described elsewhere [2]. Quality control criteria for genotyping included a call rate > 97%, missing genotype < 0.01, Hardy-Weinberg equilibrium *P* > 0.000001, and minor allele frequency > 0.01.

Anthropometric measurements were obtained by trained technicians, and lifestyle data were collected via questionnaires administered by trained interviewers. Body mass index (BMI) was calculated as weight (kg)/height (m^2), and waist circumference (WC) was measured at the midpoint between the lowest rib and the iliac crest. Blood samples were collected after at least 8 h of fasting. Alcohol consumption (g/day) was assessed based on the type, frequency, and portion size of the alcoholic beverages. Smoking status was categorized as nonsmoker, past

smoker, or current smoker. Regular exercise was defined as engaging in 30 min or more of physical activity per day.

Blood pressure (BP) was measured using standard mercury sphygmomanometers after 5 min of rest with measurements taken from both arms. Systolic blood pressure (SBP), diastolic blood pressure (DBP), and pulse rate (PR) were recorded, and the averages were used for the analysis [3]. Hypertension (HTN) was defined as SBP $\geq$ 140 mmHg, DBP $\geq$ 90 mmHg, or a prior diagnosis of HTN (PMID: 32371787). Diabetes mellitus (DM) was defined as fasting glucose $\geq$ 126 mg/dL, HbA1c $\geq$ 6.5%, or a DM diagnosis. Dyslipidemia was defined as total cholesterol (TC) $\geq$ 200 mg/dL, triglycerides (TG) $\geq$ 150 mg/dL, LDL-C $\geq$ 130 mg/dL, HDL-C $<$ 40 mg/dL (men) or $<$ 50 mg/dL (women), or a prior diagnosis.

3. Statistical Analysis

Differences between the training and validation sets were assessed using independent two-sample t-tests for continuous variables and chi-squared tests for categorical variables. Continuous variables are presented as mean ± standard deviation (SD), and categorical variables as percentages.

Principal component (PC) analysis was performed to account for potential population stratification related to sample collection regions, with the first two principal components (PC1 and PC2) included as covariates in downstream analyses. Genome-wide association analyses for PRS model development were conducted using logistic regression adjusted for age, sex, smoking status, alcohol consumption, exercise status, PC1, and PC2, as implemented in PLINK v1.9. Genome-wide significance was defined as $P < 5 \times 10^{-8}$.

Supplementary Table 1. Population characteristics for the development of the polygenic risk score model based on the Korea Genome Epidemiology Study cohort.

	**Total**	**Training Set**	**Validation Set**	***P*-value**
N	30465	21325	9140	
Female (n, %)	19416 (63.73)	13543 (63.51)	5873 (64.26)	2.18E-01
Age (years, mean± SD)	53.97±8.04	53.93±8.01	54.07±8.10	1.67E-01
Lifestyle				
**Smoking status** (n, %):				
Never	21682 (71.17)	15170 (71.14)	6512 (71.25)	
Quit	5216 (17.12)	3616 (16.96)	1600 (17.51)	1.73E-01
Current	3567 (11.71)	2539 (11.91)	1028 (11.25)	
**Drinking status** (n, %): Never/	15176 (49.81)	10615 (49.78)	4561 (49.90)	
Quit/	1149 (3.77)	796 (3.73)	353 (3.86)	8.22E-01
Current	14140 (46.41)	9914 (46.49)	4226 (46.24)	
**Exercise status** (n, %):				
No/	13855 (45.48)	9665 (45.32)	4190 (45.84)	4.11E-01
Yes	16610 (54.52)	11660 (54.68)	4950 (54.16)	
Disease				
**Hypertension** (n, %):				
No	21016 (69.00)	14738 (69.14)	6278 (68.69)	4.44E-01
Yes	9441 (31.00)	6579 (30.86)	2862 (31.31)	
**Diabetes** (n, %):				
No	25284 (82.99)	17698 (82.99)	7586 (83.00)	1.00E+00
Yes	5181 (17.01)	3627 (17.01)	1554 (17.00)	
**Obesity** (n, %):				
No	20255 (66.49)	14219 (66.68)	6036 (66.04)	2.85E-01
Yes	10210 (33.51)	7106 (33.32)	3104 (33.96)	
**Abdominal Obesity** (n, %):				
No	23269 (76.38)	16309 (76.48)	6960 (76.15)	5.45E-01
Yes	7196 (23.62)	5016 (23.52)	2180 (23.85)	
**Hypo-HDL cholesterolemia** (n, %):				
No	22312 (73.24)	15603 (73.17)	6709 (73.40)	6.81E-01
Yes	8153 (26.76)	5722 (26.83)	2431 (26.60)	
**Hypertriglyceridemia** (n, %):				
No	26349 (86.59)	18448 (86.60)	7901 (86.57)	9.57E-01
Yes	4081 (13.41)	2855 (13.40)	1226 (13.43)	
**Hypercholesterolemia** (n, %):				
No	26569 (87.21)	18610 (87.27)	7959 (87.08)	6.63E-01
Yes	3896 (12.79)	2715 (12.73)	1181 (12.92)	
**Metabolic Syndrome** (n, %):				
No	15605 (66.65)	10917 (66.70)	4688 (66.54)	8.31E-01
Yes	7808 (33.35)	5451 (33.30)	2357 (33.46)	
**Cancer** (n, %):				
No	30465 (100.00)	21325 (100.00)	9140 (100.00)	1.00E+00
Yes	0 (0.00)	0 (0.00)	0 (0.00)	
**Myocardial Infarction** (n, %):				
No	29466 (96.73)	20625 (96.74)	8841 (96.73)	1.00E+00
Yes	995 (3.27)	696 (3.26)	299 (3.27)	
**Ischemic Stroke** (n, %):				
No	30033 (98.58)	21025 (98.59)	9008 (98.56)	8.41E-01
Yes	432 (1.42)	300 (1.41)	132 (1.44)	
**Anthropometric traits**				
Body mass index (kg/m2)	23.97±2.94	23.95±2.93	24.01±2.97	1.61E-01
Waist circumference (cm)	80.89±8.77	80.87±8.78	80.94±8.76	5.15E-01
Systolic blood pressure (mmHg)	123.13±14.73	123.11±14.70	123.20±14.81	6.26E-01
Diastolic blood pressure (mmHg)	75.60±9.69	75.59±9.70	75.62±9.66	8.21E-01
Biochemical traits				
Glucose (mg/dL)	98.56±23.85	98.53±23.93	98.62±23.68	7.80E-01
HbA1C	5.71±0.72	5.72±0.73	5.71±0.70	5.04E-01
TC (mg/dL)	198.17±36.67	198.22±36.72	198.04±36.55	6.86E-01
HDL-C (mg/dL)	54.53±13.69	54.51±13.74	54.59±13.57	6.20E-01
TG (mg/dL)	128.65±90.09	128.94±90.20	127.97±89.84	3.87E-01
γ-GTP (IU/L)	31.83±42.98	31.98±44.79	31.48±38.41	3.20E-01
AST (IU/L)	23.95±28.52	24.06±33.30	23.68±11.12	1.34E-01

ALT (IU/L)	22.70±24.94	22.84±28.17	22.38±14.90	6.53E-02
ALP (IU/L)	170.52±101.28	169.93±93.65	171.89±117.19	1.66E-01
Albumin (g/dL)	4.64±0.25	4.64±0.25	4.64±0.25	8.44E-01
Uric Acid (mg/dL)	4.74±1.29	4.75±1.29	4.73±1.29	3.26E-01
Creatinine (mg/dL)	0.80±0.23	0.80±0.25	0.80±0.19	1.33E-01
BUN (mg/dL)	14.52±4.01	14.53±4.05	14.49±3.91	3.96E-01
CRP (mg/dL)	0.14±0.37	0.14±0.39	0.14±0.32	1.04E-01
WBC (Thous/uL)	5.75±1.56	5.75±1.57	5.73±1.53	1.51E-01
RBC (Mil/uL)	4.56±0.41	4.57±0.41	4.56±0.40	4.70E-01
Blood Calcium Level (mg/dL)	9.25±0.40	9.25±0.41	9.25±0.40	4.88E-01
Hemoglobin (g/dL)	13.98±1.47	13.98±1.48	13.98±1.45	8.68E-01
HCT (%)	41.76±3.79	41.76±3.81	41.76±3.74	9.40E-01
Platelet (Thous/uL)	254.53±58.86	254.52±58.75	254.56±59.14	9.54E-01
MCV (fL)	91.61±4.71	91.59±4.73	91.65±4.67	2.51E-01
MCH (pg)	30.62±1.95	30.62±1.95	30.64±1.93	3.86E-01
MCHC (g/dL)	33.42±1.05	33.42±1.05	33.41±1.05	8.99E-01

Supplementary Table 2. Distribution of patients with type 2 diabetes mellitus by polygenic risk score (PRS) model quartile group for the validation set.

Model 1	Quartile of PRS				Total	≥Q2	≥Q3	≥Q4
	Q1	Q2	Q3	**Q4**				
Total (n)	2316	2225	2374	**2225**	9140	6824	4599	2225
Control (n)	1973	1839	1962	**1812**	7586	5613	3774	1812
DM Case (n)	343	386	412	**413**	1554	1211	825	413
DM Case (%)	0.15	0.17	0.17	**0.19**	0.17	0.18	0.18	0.19
**Model 2**	**Quartile of PRS**				**Total**	**≥Q2**	**≥Q3**	**≥Q4**
	Q1	Q2	Q3	**Q4**				
Total (n)	2301	2258	2296	**2285**	9140	6839	4581	2285
Control (n)	1981	1883	1902	**1820**	7586	5605	3722	1820
DM Case (n)	320	375	394	**465**	1554	1234	859	465
DM Case (%)	0.14	0.17	0.17	**0.2**	0.17	0.18	0.19	0.2
**Model 3**	**Quartile of PRS**				**Total**	**≥Q2**	**≥Q3**	**≥Q4**
	Q1	Q2	Q3	**Q4**				
Total (n)	2742	1823	2295	**2280**	9140	6398	4575	2280
Control (n)	2325	1525	1926	**1810**	7586	5261	3736	1810
DM Case (n)	417	298	369	**470**	1554	1137	839	470
DM Case (%)	0.15	0.16	0.16	**0.21**	0.17	0.18	0.18	0.21

Model 1, Genome-wide PRS model; Model 2, East-Asian model; Model 3, HelloGene™ DM panel model. Abbreviations: DM, Diabetes

Model 1. Genome-wide model | **Model 2. East-Asian model** | **Model 3. HelloGene™ DM panel model**

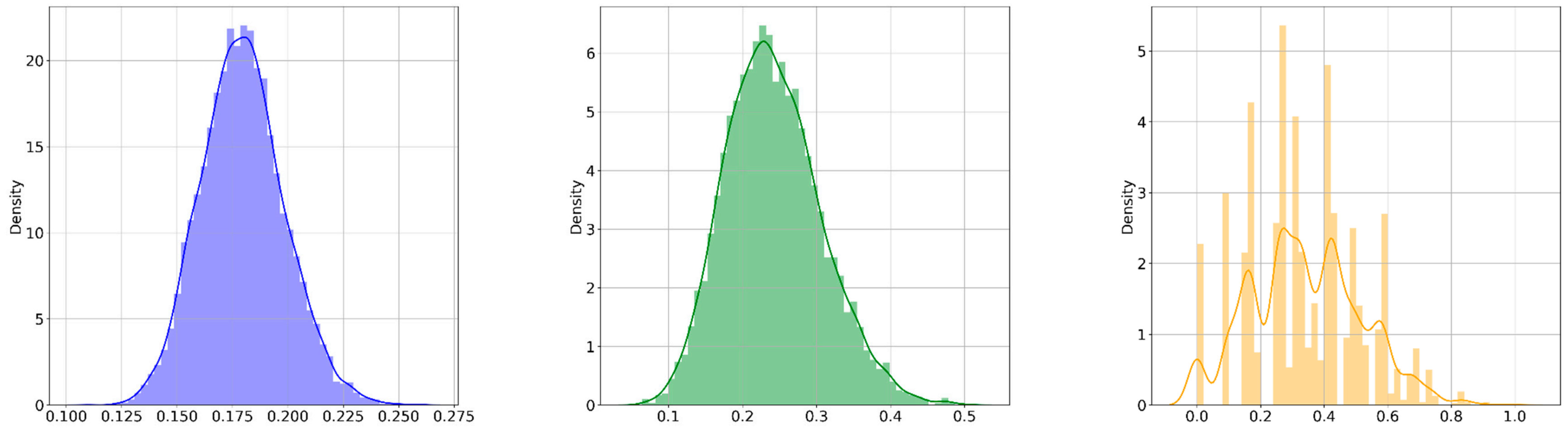

Model 1. Genome-wide model

	PRS
**N**	9140
**Mean**	0.180
**SD**	0.019
**Min**	0.110
**25%**	0.167
**50%**	0.179
**75%**	0.192
**Max**	0.259

Model 2. East-Asian model

	PRS
**N**	9140
**Mean**	0.242
**SD**	0.065
**Min**	0.065
**25%**	0.196
**50%**	0.237
**75%**	0.283
**Max**	0.504

Model 3. HelloGene™ DM panel model

	PRS
**N**	9140
**Mean**	0.341
**SD**	0.174
**Min**	0.000
**25%**	0.248
**50%**	0.335
**75%**	0.436
**Max**	1.000

Supplementary Figure 1. Distribution of three polygenic risk score models for the incidence of diabetes.

Supplementary Table 3. Frequency of type 2 diabetes mellitus-related genotypes according to HelloGene™ DM panel test quartile groups.

Target Genes	Genotypes	HelloGene^{TM} DM panel test quartile groups											
	N = non-risk allele	Low risk			Moderate risk			High risk			Very high risk		
	R = risk allele	Control (n)	DM (n)	Genotype Frequency	Control (n)	DM (n)	Genotype Frequency	Control (n)	DM (n)	Genotype Frequency	Control (n)	DM (n)	Genotype Frequency
HHEX	N/N	59	0	1.00	86	1	0.78	1	0	0.01	0	0	0.00
	N/R	0	0	0.00	24	0	0.22	67	1	0.97	30	5	0.56
	R/R	0	0	0.00	0	0	0.00	1	0	0.01	26	1	0.44
KCNQ1	N/N	26	0	0.44	30	1	0.28	36	1	0.53	27	2	0.47
	N/R	17	0	0.29	64	0	0.58	30	0	0.43	27	4	0.50
	R/R	16	0	0.27	16	0	0.14	3	0	0.04	2	0	0.03
CDKAL1	N/N	52	0	0.88	20	0	0.18	12	1	0.19	6	0	0.10
	N/R	7	0	0.12	58	1	0.53	55	0	0.79	17	2	0.31
	R/R	0	0	0.00	32	0	0.29	2	0	0.03	33	4	0.60
TCF7L2	N/N	59	0	1.00	109	1	0.99	68	1	0.99	46	4	0.81
	N/R	0	0	0.00	1	0	0.01	1	0	0.01	10	2	0.19
	R/R	0	0	0.00	0	0	0.00	0	0	0.00	0	0	0.00

Supplementary Table 4. Evaluation of clinical validity based on polygenic risk score classification criteria of the HelloGene™ DM panel test

**Statistics**	**Values above 'low risk' determined as DM**		**Values above 'moderate risk' determined as DM**		**Values above 'high risk' determined as DM**	
	**Value**	**95% CI**	**Value**	**95% CI**	**Value**	**95% CI**
**Sensitivity**	100.00%	63.06% to 100.00%	87.50%	47.35% to 99.68%	75.00%	34.91% to 96.81%
**Specificity**	20.07%	15.64% to 25.11%	57.48%	51.61% to 63.20%	80.95%	75.99% to 85.28%
**Positive likelihood ratio**	1.25	1.18 to 1.32	2.06	1.53 to 2.76	3.94	2.47 to 6.26
**Negative likelihood ratio**	0		0.22	0.03 to 1.36	0.31	0.09 to 1.03
**Disease prevalence**	8.00%		8.00%		8.00%	
**Positive predictive value**	9.81%	9.32% to 10.33%	15.18%	11.77% to 19.36%	25.51%	17.71% to 35.26%
**Negative predictive value**	100.00%	93.94% to 100.00%	98.14%	89.40% to 99.70%	97.38%	91.80% to 99.20%
**Accuracy**	26.46%	21.57% to 31.82%	59.88%	54.12% to 65.46%	80.48%	75.55% to 84.79%

References

1. Kim, B., et al., *The cut-off values of surrogate measures for insulin resistance in the Korean population according to the Korean Genome and Epidemiology Study (KOGES).* PLoS One, 2018. **13**(11): p. e0206994.
2. Moon, S., et al., *The Korea Biobank Array: Design and Identification of Coding Variants Associated with Blood Biochemical Traits.* Sci Rep, 2019. **9**(1): p. 1382.
3. Lim, N.K., et al., *Predicting the risk of incident hypertension in a Korean middle-aged population: Korean genome and epidemiology study.* J Clin Hypertens (Greenwich), 2013. **15**(5): p. 344-9.
